# Research on the Physical Behaviors of AG-80 Epoxy Resins: Moisture, Thermal, and Mechanical Insights

**DOI:** 10.3390/polym17060707

**Published:** 2025-03-07

**Authors:** Guancheng Chen, Jian Yu, Xin Xiong, Zhenxing Wang, Jiawei Wu, Xinfeng Wang, Shuo Huang

**Affiliations:** 1 College of Aerospace Engineering, Nanjing University of Aeronautics and Astronautics, Nanjing 210016, China; chenguancheng0616@163.com (G.C.); wujiawei@nuaa.edu.cn (J.W.); xinfengw@nuaa.edu.cn (X.W.); 2 College of General Aviation and Flight, Nanjing University of Aeronautics and Astronautics, Nanjing 210016, China; 3 China Helicopter Research and Development Institute, Tianjin 300308, China; xiongx005@avic.com (X.X.); 18729012937@163.com (Z.W.); 4 College of Information Science and Technology, Nanjing Forestry University, Nanjing 210037, China; huangshuo@njfu.edu.cn

**Keywords:** epoxy resins, DMA, CFRP, moisture absorption, constitutive model

## Abstract

This study focuses on AG-80 epoxy resin, using 5228A and BA9916-II as representative examples. Saturated moisture absorption tests and dynamic mechanical analyses (DMA) were conducted to investigate its physical properties. The saturated moisture absorption rates and diffusion coefficients for 5228A and BA9916-II were measured. Their distinct molecular structures were found to lead to different moisture–absorption behaviors. A hygro-thermal–mechanical constitutive model for AG-80 resin (represented by 5228A) was developed, taking into account the effects of temperature and moisture content on mechanical properties. The model was validated by DMA tests on 5228A/CCF300 composites. The research findings of this study enhance the understanding of AG-80 epoxy resin and provide a theoretical basis for its application in high-temperature-resistant industrial environments.

## 1. Introduction

In modern high-tech industries such as aerospace, automotive, and microelectronics, materials with exceptional high-temperature resistance play a crucial role. Epoxy resins are renowned for their favorable mechanical properties, strong adhesion, and excellent chemical resistance, making them essential materials in these fields [1,2,3]. Among them, the AG-80 epoxy resin is considered a promising candidate for high-temperature applications due to its unique molecular structure and composition [4,5].

The AG-80 epoxy resin exists in various types, with 5228A and BA9916-II being two representative variants. These two types have attracted significant attention for their potential to withstand high-temperature environments. In aerospace applications, components like the leading edges of hypersonic aircraft are exposed to extreme aerodynamic heating, where temperatures can reach several hundred degrees Celsius. In the automotive industry, as engines aim for higher efficiency, the under-hood temperature continues to rise. In both cases, materials with reliable high-temperature performance are needed to ensure the long-term stability and functionality of components. AG-80 epoxy resin, especially its 5228A and BA9916-II types, has the potential to meet these stringent requirements.

However, when applying AG-80 epoxy resin in practical scenarios, several challenges arise. The curing process of this resin is highly complex and sensitive to temperature. Incorrect curing conditions can lead to incomplete cross-linking, which not only deteriorates the mechanical properties but also reduces its high-temperature resistance [6]. Additionally, during the curing process, significant volumetric shrinkage occurs, which may introduce internal stress and cause deformation in the final product [7]. These issues can compromise the dimensional accuracy and structural stability of components made from AG-80 epoxy resin.

To address these challenges, a comprehensive understanding of physical properties, including moisture–absorption characteristics, thermal–mechanical behaviors, and the influence of environmental factors such as temperature and humidity, is essential. Studying moisture–absorption properties helps in predicting the resin’s performance in humid environments, which is crucial for applications where the material may be exposed to moisture. Analyzing the mechanical properties under different thermal conditions can provide insights into its behavior during service in high-temperature environments.

In this research, we utilize 5228A and BA9916-II as examples to conduct in-depth investigations on the AG-80 epoxy resin. Through a series of experimental methods such as saturated moisture absorption tests and dynamic mechanical analysis (DMA), we aim to systematically study its physical properties. The specific objectives include determining the saturated moisture absorption rates and diffusion coefficients, analyzing the viscoelastic properties, and establishing a hygro-thermal–mechanical constitutive model. The results of this study will contribute to a better understanding of the AG-80 epoxy resin and offer valuable guidance for its practical applications in high-temperature-demanding industries.

## 2. Experiment Essentials

### 2.1. Materials

Two new types of AG-80 resins, namely 5228A and BA9916-II, were synthesized by AVIC Composite Corporation LTD. Both resins are cured with DDS (diaminodiphenyl sulfone) as the curing agent. However, they differ in their epoxy functional group content; 5228A has a lower epoxy content, resulting in a higher cross-linking density and fewer polar groups, while BA9916-II contains more epoxy groups, leading to a higher density of polar functional groups (e.g., hydroxyl and carbonyl) and more flexible molecular chains. The resin-to-curing agent ratio for both 5228A and BA9916-II was optimized according to their respective epoxy contents to ensure complete curing and desired mechanical properties. The corresponding curing agents for 5228A and BA9916-II were used as per the manufacturer’s recommended formulations [8]. Accurate control of the ratio of resin to curing agent is crucial for proper curing and desired material properties [9].

In addition, high-modulus carbon fibers CCF300, sourced from AVIC Composite Corporation LTD, were utilized to fabricate fiber-reinforced composites with the AG-80 resins (5228A and BA9916-II) to further investigate the mechanical and thermal properties of the resulting composites during the curing process.

### 2.2. Instruments and Equipment

A diverse set of specialized instruments was used for comprehensive tests of physical properties on AG-80 resins (5228A and BA9916-II) and their composites. The TA Instruments DMA850 was used for thermal dynamic mechanical analysis (DMA) to measure the viscoelastic properties of materials during curing, thereby determining the storage modulus, loss modulus, and damping factor as functions of temperature and frequency [10]. The MTS15000 was used to test cured resins and composites in tensile tests, providing stress–strain data for the calculation of tensile strength and elastic modulus. The TA Instruments Discovery HR-2 rheometer was used for rheological tests, measuring resin viscosity changes with temperature, shear rate, and time to determine gel time and understand processing flow behavior. The CTHSG4050C-02F programmable temperature and humidity chamber was used in saturated moisture absorption tests to control environmental conditions. The weight change in specimens was measured to calculate the saturated moisture absorption rate. The chemical structure of the resins was monitored in real-time during the curing process using a Nicolet iS50 Fourier transform infrared (FTIR) spectrometer, which detected the absorption of infrared light. An XS105DU analytical balance measured the density of resins and composites, and the CQ-J12 curemeter accurately measured the resins’ gel time.

### 2.3. Specimen Preparation and Testing

The well-mixed AG-80 resins (5228A and BA9916-II) were first placed in a vacuum drying oven for degassing and then transferred into molds for curing as per the following recommended process: 24 h at room temperature followed by 8 h of post-curing at 80 °C.

DMA tests on both resins and composites were conducted in accordance with ASTM D7028-07. The cured resin blocks were cut and polished into specimens sized 10 mm × 50 mm × 3 mm, and composite specimens were made by laying up unidirectional prepregs, curing, and then cutting them to the same dimensions. The tests were conducted at 23 °C and 35% humidity, with a 5 °C/min heating rate, a 1 Hz frequency, and a dual-cantilever fixture. Specimens were either dry or wet, with wet specimens prepared by immersion in 71 °C water for 14 days.

The cured resin blocks were cut and polished into 15 mm × 250 mm × 6 mm specimens for tensile tests. Resin castings were used as test specimens, and tensile strength, tensile modulus, and elongation at break were measured according to ASTM D638-2014 to ensure reliable results.

Solid specimens were used in rheological tests. Under environmental conditions of 20 °C and 50% humidity, a programmed heating process was applied, gradually increasing the temperature of the specimens from 3 °C to 200 °C at a 2 °C/min rate to evaluate rheological characteristics.

## 3. Results and Discussion

### 3.1. Moisture Absorption Tests on AG-80 Resins

The saturated moisture absorption tests were conducted in strict accordance with the ASTM D5229/D5229M-14 standards [11]. Initially, the specimens were placed in an environment where the temperature was maintained at 22 °C and the relative humidity was 58%. After that, they were transferred to another environment with a temperature of 71 °C and a relative humidity of 85%. The specimens were kept in this environment until moisture absorption equilibrium was achieved.

**Figure 1:**
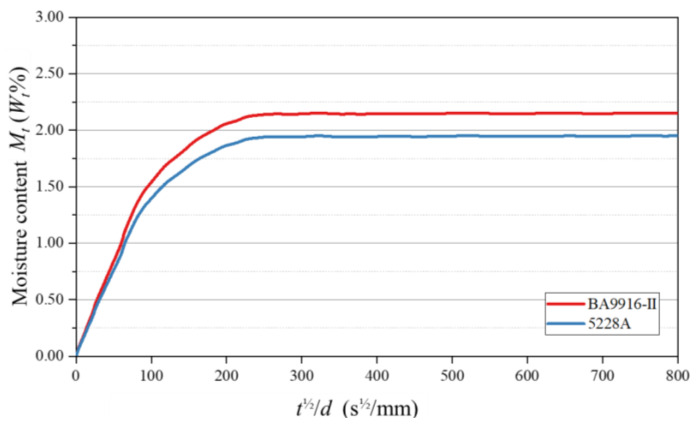
Moisture absorption curves of AG-80 resin.

Through the water-immersion moisture absorption tests, the moisture absorption levels of the two resins at different time points were measured. Fick’s diffusion law [12] is expressed as follows:

(1) $$\frac{M_{t}}{M_{\infty }}=4{\left(\frac{Dt}{\pi d^{2}}\right)}^{\frac{1}{2}}$$

where $M_{t}$ and $M_{\infty }$ represent the moisture contents of the specimen at time t and at equilibrium, respectively; D is the diffusion coefficient; and d is the specimen thickness. By plotting $M_{t}$ against t12/d, the moisture absorption curve of the specimen was obtained (as shown in Figure 1). From the horizontal extension line and the linear part of the curve, $M_{\infty }$ and D could be determined, respectively, and the values are listed in Table 1.

**Figure 2:**
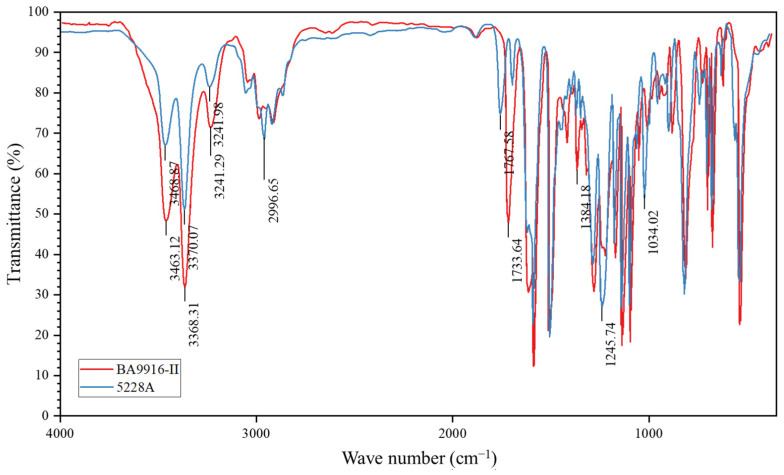
Infrared spectra of AG-80 resin.

This difference results from their distinct molecular structures, as supported by the analysis of infrared spectra. Figure 2 shows the infrared spectra of AG-80 resin, which indicate that BA9916-II shows significantly lower transmittance (more than 20% lower) than 5228A at approximately 1717 cm^−1^, 3360 cm^−1^, and 3465 cm^−1^. The absorption peak at 1717 cm^−1^ is attributed to the stretching vibration of the carbonyl (C=O) group. This implies that BA9916-II likely has a higher content of carbonyl-containing functional groups such as ketones or conjugated carbonyls. Meanwhile, the absorption peaks at 3360 cm^−1^ and 3465 cm^−1^ are related to the stretching vibrations of N-H or O-H groups, suggesting that BA9916-II contains more alcohol hydroxyl, phenolic hydroxyl, amine, or amide structures.

5228A has a relatively low density of polar functional groups. Polar groups such as hydroxyl (-OH), amino (-NH_2_), and carbonyl (C=O) groups exhibit strong affinity towards water molecules through hydrogen-bonding interactions. With a lower density of these polar groups, as also evidenced by the relatively higher transmittance in the corresponding infrared regions, there are fewer sites available for water molecule adsorption, resulting in a lower saturated moisture absorption rate. Additionally, the molecular chains of 5228A exhibit higher cross-linking density and a more compact packing arrangement. A high degree of cross-linking restricts the mobility of molecular chains and reduces the free volume within the material, thereby impeding the diffusion of water molecules and limiting moisture absorption.

In contrast, BA9916-II has more polar groups, as indicated by the strong infrared absorption in the regions related to polar functional groups. These additional polar groups provide more sites for hydrogen bonding with water molecules. Moreover, it has more flexible chains, which provide less hindrance to the movement of water molecules. This combination facilitates water diffusion and leads to higher moisture absorption. These results are consistent with previous studies on similar epoxy resins, such as the moisture absorption behavior reported by Xu et al. [4] for T800/high-temperature epoxy composites.

### 3.2. DMA Tests on AG-80 Resins

**Figure 3:**
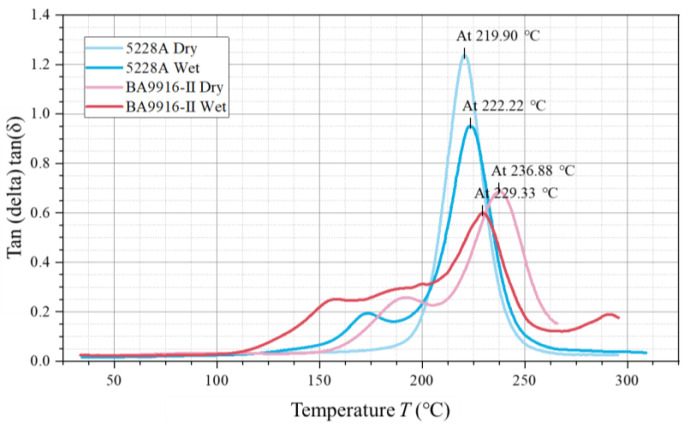
The curve of the loss factor of AG-80 resin vs. temperature.

Four loss factor curves were obtained (in Figure 3), representing dry 5228A resin, wet 5228A resin, dry BA9916-II resin, and wet BA9916-II resin. Each curve exhibits a single peak characteristic that represents the glass transition process of the materials. The peak temperature of the dry 5228A resin curve is 219.90 °C and has a maximum loss factor of approximately 1.2. For the wet 5228A resin, the peak temperature rises to 222.22 °C, while the loss factor decreases to around 0.95. The dry BA9916-II resin curve has a peak temperature of 236.88 °C and a loss factor of about 0.7. In the case of the wet BA9916-II resin, the peak temperature drops to 229.33 °C, and the loss factor reaches the lowest value of approximately 0.6.

**Figure 4:**
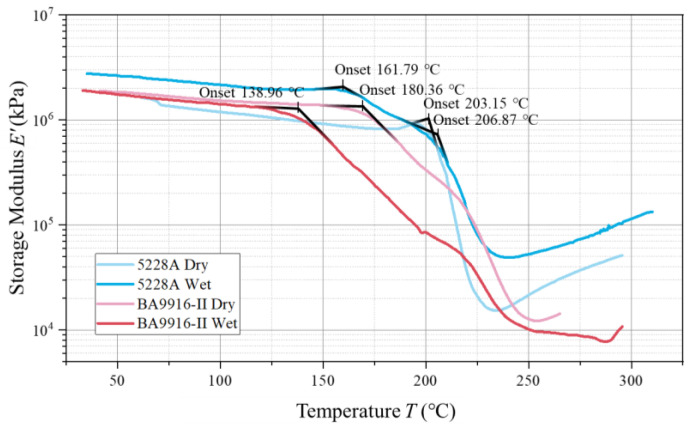
The curve of the storage modulus of AG-80 resin vs. temperature.

In the storage modulus curves (in Figure 4), the onset temperature of the slope change reflects the starting point when the material’s properties begin to change significantly. The onset temperature of the dry 5228A resin is 203.15 °C. The wet 5228A resin shows two slope changes at 161.79 °C and 206.87 °C, respectively. Both the dry and wet BA9916-II resins have an onset temperature of 180.36 °C. Notably, for the storage modulus, the dry-state 5228A resin has a value around 10^6^ kPa, with a significant increase in the wet state. In contrast, for BA9916-II resin, the change in storage modulus between the dry and wet states is not obvious, only showing a change in the onset temperature.

**Figure 5:**
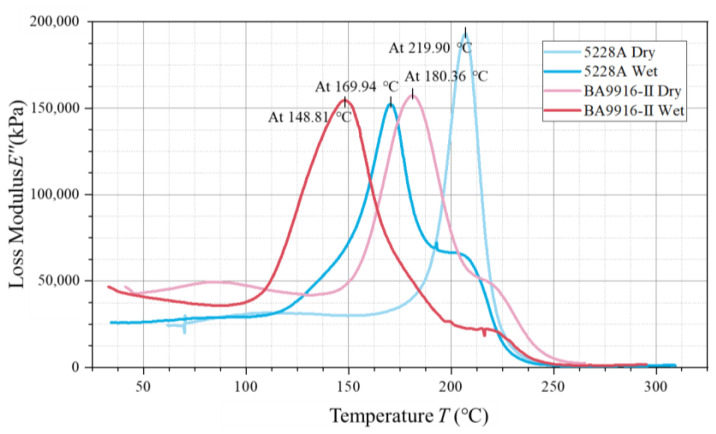
The curve of the loss modulus of AG-80 resin vs. temperature.

The loss modulus curves also have peak characteristics (in Figure 5), where the peak temperature corresponds to the activated state of the internal molecular motion of the material. The dry 5228A resin has a peak temperature of 219.90 °C, with a loss modulus of approximately 190,000 kPa. For the wet 5228A resin, the peak temperature drops to 169.94 °C, and the loss modulus is about 150,000 kPa. The dry BA9916-II resin has a peak temperature of 180.36 °C, and the wet BA9916-II resin has a peak temperature of 148.81 °C, with both having a loss modulus of around 160,000 kPa.

In the dry state, the $T_{g}$ of BA9916-II resin (236.88 °C) is significantly higher than that of 5228A resin (219.90 °C). This indicates that the molecular segments of BA9916-II resin require more energy for movement. Its molecular structure may be more rigid, with a higher degree of cross-linking, more hydrogen bonds, or other strong intermolecular forces [13]. After moisture absorption, the $T_{g}$ of 5228A resin shows a slight increase. This is due to the formation of hydrogen bonds between water molecules and polar groups, which enhances intermolecular forces. However, the decrease in loss factor is due to the lubricating effect of water molecules, which reduces direct friction between molecular chains. In contrast, the $T_{g}$ of BA9916-II resin decreases, suggesting that water molecules act as a plasticizer, weakening the intermolecular forces and facilitating the movement of molecular segments.

The loss factor of dry 5228A resin is higher than that of BA9916-II resin, indicating that 5228A resin exhibits higher energy dissipation during deformation. This is because the internal friction between its molecular chains is relatively large, resulting in the movement of molecular chains being more disordered. After moisture absorption, the loss factors of both resins decrease. This is because the presence of water molecules reduces direct friction between molecular chains, thereby decreasing energy dissipation.

In the storage modulus curves, the onset temperature of dry 5228A resin is higher than that of dry BA9916-II resin, which is consistent with the $T_{g}$ difference reflected in the loss factor curves. This further demonstrates that 5228A resin starts to show significant property changes at a higher temperature, indicating better thermal stability. The wet 5228A resin shows two slope changes, which may imply that there are two different structural change processes inside the resin after moisture absorption. The first change may be related to the interaction between water molecules and some polar groups, while the second change may correspond to the overall change in the movement of molecular segments. In contrast, the onset temperature of BA9916-II resin remains the same in both dry and wet states, indicating that moisture absorption has little effect on the starting temperature at which its properties begin to change.

The significant increase in the storage modulus of 5228A resin after moisture absorption suggests that water molecules may interact with the resin matrix by enhancing the material’s ability to store elastic energy. The interaction between water molecules and the resin is complex. In 5228A resin, it is manifested as enhancing the intermolecular forces to a certain extent and increasing the storage modulus. However, this does not mean that the overall rigidity is enhanced; it only changes in terms of elastic energy storage. For BA9916-II resin, this interaction is mainly reflected in changing the forces between molecular chains, increasing its flexibility, and having a minor effect on elastic energy storage. For BA9916-II resin, the minimal change in storage modulus indicates that its molecular structure is relatively less affected by moisture absorption in terms of elastic energy storage. The change in onset temperature, nevertheless, still shows that moisture can influence the temperature at which the material’s viscoelastic properties start to change.

In the loss modulus curves, in the dry state, the peak temperature of the loss modulus of 5228A resin is higher than that of BA9916-II resin, which again proves that the molecular segments of 5228A resin require more energy to move. After moisture absorption, the peak temperatures of the loss modulus of both resins decrease, which is consistent with the change trend of $T_{g}$ after moisture absorption, indicating that water molecules promote the movement of molecular segments. The peak loss modulus of dry 5228A resin is higher than that of the wet state and also higher than that of BA9916-II resin. This indicates that the internal molecular motion of dry 5228A resin is more intense during the glass transition process, resulting in greater energy dissipation. The loss moduli of dry and wet BA9916-II resins are similar, suggesting that moisture absorption has a minor effect on its energy dissipation during the glass transition process. The observed $T_{g}$ values are higher than those of conventional DGEBA-based epoxy resins [14], indicating the superior thermal stability of AG-80 epoxy resin.

### 3.3. Development of a Hygro-Thermal Constitutive Model for AG-80 Resin

In dynamic mechanical analysis (DMA) of resin materials, understanding the viscoelastic behavior under specific conditions is crucial. Given that the tests were conducted at a fixed frequency of 1 Hz, this section presents a generalized Maxwell-based constitutive model that accounts for the influence of temperature and moisture absorption on the resin’s mechanical properties. The generalized Maxwell model serves as the foundation for describing the viscoelastic characteristics of the resin. It comprises multiple Maxwell elements connected in parallel, in which each Maxwell element consists of a spring, representing elastic behavior, and a dashpot, representing viscous behavior, connected in series.

At a fixed angular frequency ω=2π rad/s, corresponding to a frequency of 1 Hz, the storage modulus $E^{\prime }$ and loss modulus $E^{\prime \prime }$ of the resin can be described by the following equations:

(2) $$E^{\prime }=E_{0}+\overset{n}{\underset{i=1}{\sum }}E_{i}\frac{{\left(2\pi \right)}^{2}\tau_{i}^{2}}{1+{\left(2\pi \right)}^{2}\tau_{i}^{2}}$$

(3) $$E^{\prime \prime }=\overset{n}{\underset{i=1}{\sum }}E_{i}\frac{2\pi \tau_{i}}{1+{\left(2\pi \right)}^{2}\tau_{i}^{2}}$$

(4) $$\tan\delta =\frac{E^{\prime \prime }}{E^{\prime }}$$

where $E_{0}$ is the instantaneous elastic modulus, $E_{i}$ is the spring modulus of the ith Maxwell element, and $\tau_{i}$ is the relaxation time of the ith Maxwell element.

To incorporate the effects of moisture absorption, we assume that the relationship between the modulus, moisture content, and temperature is as follows. The instantaneous elastic modulus $E_{0}$ and the spring moduli $E_{i}$ of each Maxwell element are functions of moisture content m and temperature T. They can be expressed as follows:

(5) $$E_{0}\left(m,T\right)=E_{00}f_{0}\left(m,T\right)$$

(6) $$E_{i}\left(m,T\right)=E_{i0}f_{i}\left(m,T\right)$$

where $E_{00}$ and $E_{i0}$ are the instantaneous elastic modulus and the spring modulus of the ith Maxwell element in the dry state, respectively. $f_{0}\left(m,T\right)$ and $f_{i}\left(m,T\right)$ are functions of moisture content m and temperature T. Polynomial forms can be used to represent, for example, the following functions:

(7) $$f_{0}\left(m,T\right)=a_{00}+a_{01}m+a_{02}T+a_{03}mT+\cdots$$

(8) $$f_{i}\left(m,T\right)=a_{i0}+a_{i1}m+a_{i2}T+a_{i3}mT+\cdots$$

The coefficients $a_{ij}$ need to be determined by fitting the experimental data.

Regarding the relationship between relaxation time, moisture content, and temperature, the relaxation time $\tau_{i}$ of the ith Maxwell element can be similarly expressed as follows:

(9) $$\tau_{i}\left(m,T\right)=\tau_{i0}g_{i}\left(m,T\right)$$

where $\tau_{i0}$ is the relaxation time of the ith Maxwell element in the dry state, and $g_{i}\left(m,T\right)$ is a function of moisture content m and temperature T. The following polynomial form can also be used:

(10) $$g_{i}\left(m,T\right)=b_{i0}+b_{i1}m+b_{i2}T+b_{i3}mT+\cdots$$

The least-squares method is applied to fit the 5228A resin constitutive model. First, experimental data of storage modulus, loss modulus, and loss factors are obtained from DMA tests under different temperature and moisture conditions. An objective function is defined to minimize the squared differences between experimental and predicted results. Initial values are assigned for unknown model parameters. An optimization algorithm iteratively adjusts these parameters until the objective function converges, yielding the fitted parameters. This behavior aligns with findings from Rooney et al. [15], who reported similar temperature-dependent viscoelastic properties in 3D-printed resin composites.

### 3.4. Validation of Constitutive Model via 5228A/CCF300 DMA Tests

Following the establishment of the constitutive model for 5228A resin that accounts for temperature and moisture absorption effects, the model is extended to composite materials. A simple rule-of-mixtures approach is adopted to estimate the viscoelastic properties of the composites. This method offers a simple approach to combine the properties of the individual components to predict the overall behavior of the composite.

The storage modulus $E_{c}^{\prime }$ of the composite is calculated as a weighted sum of fiber and resin contributions. It is calculated as $E_{c}^{\prime }=V_{f}E_{f}+\left(1-V_{f}\right)E^{\prime }$, where $V_{f}$ is the fiber volume fraction, $E_{f}$ is the modulus of fiber, and $E^{\prime }$ is the resin storage modulus determined by the resin-based model.

Since fibers do not contribute to energy dissipation, the composite’s loss modulus $E_{c}^{\prime \prime }$ is primarily derived from the resin matrix and is expressed as $E_{c}^{\prime \prime }=\left(1-V_{f}\right)E^{\prime \prime }$, with $E^{\prime \prime }$ being the resin loss modulus.

**Figure 6:**
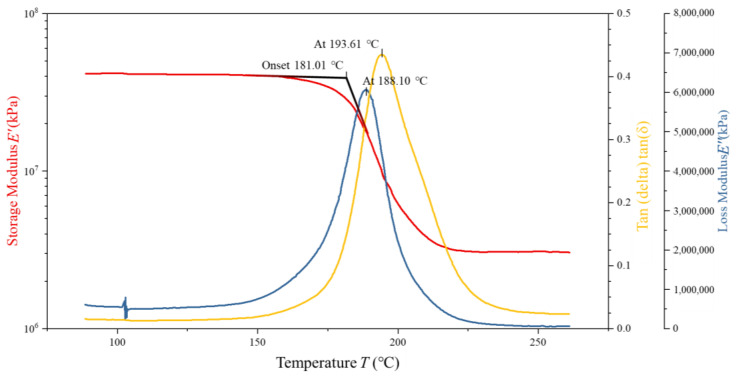
DMA test curves of dry 5228A/CCF300.

**Figure 7:**
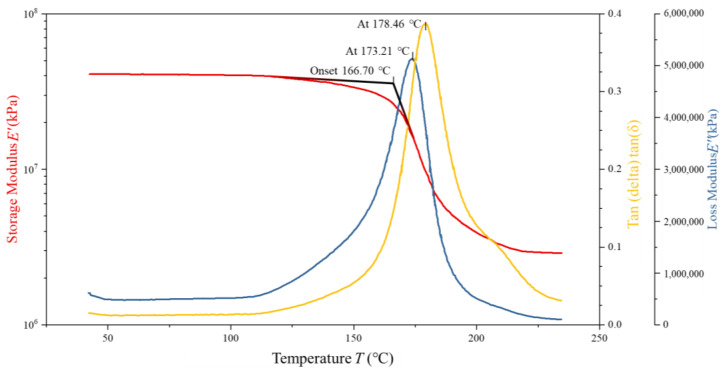
DMA test curves of wet 5228A/CCF300.

To assess the validity and predictive accuracy of the proposed 5228A resin constitutive model, DMA tests were conducted on four 5228A/CCF300 composite specimens with dimensions of 35 mm × 10 mm × 2 mm and a fiber volume fraction of 16.6667%. Tensile tests indicated that the 5228A resin had a tensile modulus of 3.22 GPa. Two specimens were in a dry state and two in a wet state. The CCF300 carbon fibers in these composites exhibited a tensile modulus of 235 GPa. The analysis of the resulting DMA curves serves as a basis for comparing the model predictions with experimental results. The DMA curves of one dry-state and one wet-state specimens are shown in Figure 6 and Figure 7.

**Figure 8:**
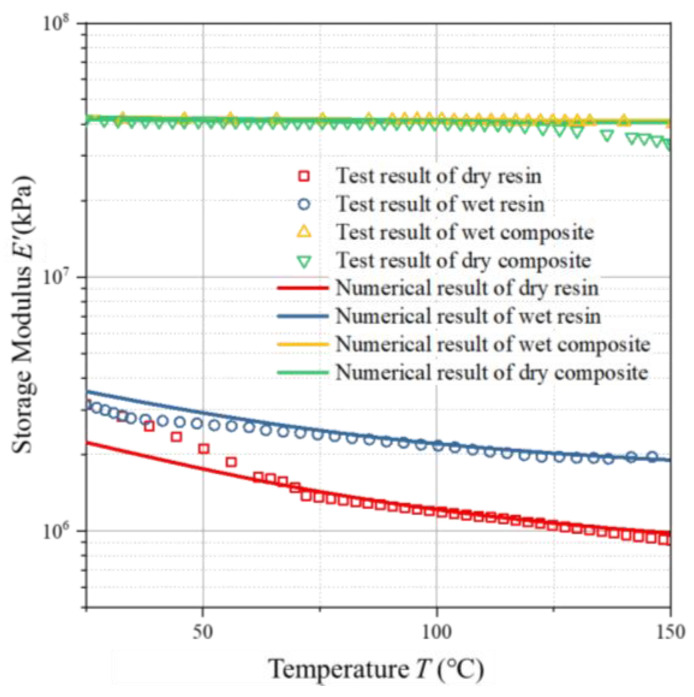
Predicted vs. experimental storage moduli of AG-80 resin and composites vs. temperature.

The comparison results between the model predictions and the measured values are shown in Figure 8. As can be seen from this figure, for the 5228A resin, the storage modulus $E^{\prime }$ decreases with the increase in temperature. The model prediction results are consistent with the experimental results. The viscoelastic model derived from the generalized Maxwell model can well reflect the properties of the material as an elastic solid and a viscous fluid.

## 4. Conclusions

In this study, a comprehensive investigation into the physical behaviors of AG-80 epoxy resin, using 5228A and BA9916-II as representative samples, was successfully conducted. Through saturated moisture absorption tests, the saturated moisture absorption rates and diffusion coefficients of the two resins were accurately determined. The differences in their molecular structures were identified as the key factors contributing to their distinct moisture–absorption behaviors. Specifically, BA9916-II, with its higher number of polar groups and more flexible molecular chains, exhibited a greater saturated moisture absorption rate compared to 5228A.

DMA tests provided in-depth insights into the viscoelastic properties of the resins. The glass transition temperatures, loss factors, storage moduli, and loss moduli of both dry and wet 5228A and BA9916-II resins were analyzed. The results revealed that moisture absorption had differing effects on the viscoelastic properties of the two resins. For instance, the glass transition temperature of 5228A resin slightly increased after moisture absorption, whereas that of BA9916-II resin decreased.

A hygro-thermal constitutive model for 5228A resin was developed, incorporating the effects of temperature and moisture content on its mechanical properties. This model was further extended to composite materials using a simple rule-of-mixtures approach. Validation through DMA tests on 5228A/CCF300 composites demonstrated that the model could effectively describe the viscoelastic behavior of both the resin and composite materials under varying conditions. The predicted storage modulus decreased with increasing temperature, which aligned well with the experimental results.

Based on research, 5228A resin is more advantageous in high-temperature, humidity-sensitive applications needing high rigidity and dimensional stability. Its low moisture absorption rate and increased $T_{g}$ after absorption help maintain component accuracy and stability, like in aerospace precision parts. BA9916-II resin, despite higher moisture absorption and decreased $T_{g}$ after absorption, has a high dry-state $T_{g}$ and flexible molecular chains. It is more suitable for applications needing some flexibility with acceptable humidity and temperature impacts, such as certain high-temperature sealing materials.

Overall, this research advances the understanding of the physical behaviors of AG-80 epoxy resin. The findings provide a theoretical foundation for optimizing the processing parameters of AG-80 resin and its composites in high-temperature-resistant applications. Future research can enhance the model in this study in several ways. First, optimize model parameters for different AG-80 epoxy resin variants and their mixtures with other epoxy resins to better predict the performance of various resin systems. Additionally, study the impact of different humidity change rates on resin performance and integrate this into the model for more accurate descriptions under complex conditions. Finally, for AG-80-based composites, focus on their long-term performance under cyclic temperature, humidity, and mechanical loads in service environments, providing a theoretical basis for long-term reliability assessments in high-demand engineering applications.

## Figures and Tables

**Table 1:** Diffusion coefficient and equilibrium moisture content of AG-80 resin.

	$M_{\infty }$ (*W_t_*%)	D (mm^2^/s)
5228A	1.95	3.08 × 10^−6^
BA9916-II	2.15	3.14 × 10^−6^

## Data Availability

The original contributions presented in this study are included in the article. Further inquiries can be directed to the corresponding author(s).
